# The Prevalence of Insomnia and the Link between Iron Metabolism Genes Polymorphisms, *TF* rs1049296 C>T, *TF* rs3811647 G>A, *TFR* rs7385804 A>C, *HAMP* rs10421768 A>G and Sleep Disorders in Polish Individuals with ASD

**DOI:** 10.3390/ijerph17020400

**Published:** 2020-01-08

**Authors:** Karolina Skonieczna-Żydecka, Dominika Jamioł-Milc, Krzysztof Borecki, Ewa Stachowska, Paulina Zabielska, Magdalena Kamińska, Beata Karakiewicz

**Affiliations:** 1 Department of Human Nutrition and Metabolomics, Pomeranian Medical University in Szczecin, 71-460 Szczecin, Poland; karzyd@pum.edu.pl (K.S.-Ż.); dominikajamiol@interia.pl (D.J.-M.); k.borecki.pum@gmail.com (K.B.); ewast@pum.edu.pl (E.S.); 2 Department of Social Medicine and Public Health, Chair of Social Medicine, Pomeranian Medical University in Szczecin, 71-210 Szczecin, Poland; beata.karakiewicz@pum.edu.pl; 3 Subdepartment of Long Term Care, Chair of Social Medicine, Pomeranian Medical University in Szczecin, 71-210 Szczecin, Poland; magdalena.kaminska@pum.edu.pl

**Keywords:** autism spectrum disorders, polymorphism, insomnia

## Abstract

Iron deficiency have been found to be linked to sleep disorders. Both genetic and environmental factors are risk factors for skewed iron metabolism, thus sleep disruptions in autism spectrum disorders (ASD). The aim of our study was to assess the prevalence of single nucleotide polymorphisms (SNPs) within transferrin gene (*TF)* rs1049296 C>T, rs3811647 G>A, transferrin receptor gene (*TFR)* rs7385804 A>C, and hepcidin antimicrobial peptide gene (*HAMP)* rs10421768 A>G in Polish individuals with ASD and their impact on sleep pattern. There were 61 Caucasian participants with ASD and 57 non-ASD controls enrolled. Genotypes were determined by real-time PCR using TaqMan SNP assays. The Athens Insomnia Scale (AIS) was used to identify sleep disruptions. There were 32 cases (57.14%) with insomnia identified. In the ASD group, the defined counts of genotypes were as follows: *TF* rs1049296, C/C *n =* 41 and C/T *n =* 20; *TF* rs3811647, G/G *n =* 22, G/A *n =* 34, and A/A *n =* 5; *TFR* rs7385804, A/A *n =* 22, A/C *n =* 29, and C/C *n =* 10; and *HAMP* rs10421768, A/A *n =* 34, A/G *n =* 23, and G/G *n =* 4. There were no homozygous carriers of the *TF* rs1049296 C>T minor allele in the ASD group. All analyzed SNPs were not found to be linked to insomnia. The investigated polymorphisms are not predictors of sleep disorders in the analyzed cohort of individuals with ASD.

## 1. Introduction

Autism spectrum disorders (ASD) belong to the group of pervasive developmental disorders of childhood. Clinical characteristics include: (1) deficits in social interaction; (2) impaired ability to communicate; and (3) behavior marked by stereotypies and narrow repertoire of activity [1,2]. The triad of symptoms manifests itself in early childhood, usually before 36 months of age (infantile autism) and continues throughout life [1,3]. According to the fifth Diagnostic and Statistical Manual of psychiatric disorders (DSM-V) released in 2013 by the American Association of Psychiatrists, culminating a 14-year revision process, all prior diagnosis of autism, unspecific comprehensive development disorder, Asperger’s syndrome, and childhood disintegrative disorders are now collectively referred to ASD [4]. According to the Center for Disease Control and Prevention (CDC), one in 59 children is estimated to have autism. In Poland, the prevalence of ASD in children aged 0–16 years varies between two regions of Poland—32/10,000 in West Pomeranian and 38/10,000 in Pomeranian region [5]. ASD is accompanied by mental retardation in three out of four patients, and boys are four times more likely than girls to receive the diagnosis [6]. The underlying etiology involves the interaction of multiple genetic and environmental factors [7].

Somatic and psychological comorbidities in ASD are common, explaining the complexity of the disorder etiology [7]. One of the most abundant are sleep problems affecting as much as 80% of autistic individuals [8]. The alterations include insomnia manifested by parasomnia, breathing disorders, bedtime resistance, long sleep latency, and daytime somnolence and irritability [9]. As sleep was proved to be essential for human nervous system functioning, especially during childhood when brain growth takes place intensively [10], sleep disorders in early life negatively correspond to physical and mental health. They affect behavior and social identification and cause irritability, mood swings, aggression, and globally motional distresses [11]. In fact, the direct association between sleep problems and autistic behavior was proved [12]. Moreover, sleep fragmentation increases the risk for long-term adverse outcomes, especially of cardiovascular, neurological, and cognitive nature. Metabolic and endocrine impairments being predominantly correlated to respiratory-related sleep disorders are potential comorbidities [13].

The genetic background of sleep difficulties include polymorphism within biological clock genes controlling circadian rhythm and melatonin synthesis and secretion [14,15,16,17], predominantly. On the other hand, there is evidence about the essential impact of iron on cognitive thus behavioral development [18,19]. Indeed, iron is critical for a myelinogenesis and is a component of enzymes involved in synthesis and modes of action of monoamine neurotransmitters, namely tryptophan hydroxylase (serotonin) and tyrosine hydroxylase (noradrenalin and dopamine) [20]. In vivo studies proved that lack of iron is associated with a decrease in extracellular concentrations of dopamine and reduction of the density of the dopamine receptors and transporters in the brain [21]. Moreover, it was shown that the human sleep–wake cycle is at least partly controlled by the dopamine-opiate system [22]. Both in vitro and in vivo models of iron insufficiency suggested that iron and dopamine may be crucial for sleep disorders [23,24]. These all make iron and genes involved in this trace element metabolism as targets in genetic analyses of comorbidities associated with autistic behavior. It seems even more obvious, as it was proved, that low serum ferritin level [25] and reduced iron concentration in the cerebrospinal fluid and brain may significantly delay the time to fall asleep and they are associated with the severity of the restless legs syndrome [26,27]. Other researchers have confirmed that sleep disorders in patients with ASD, i.e., long sleep latency, multiple awakenings during night, and increased muscle activity during rapid eye movement (REM) phase, are alleviated during iron supplementation [28]. These confirm the existence of a relationship between the normal concentration of this element and uninterrupted sleep architecture and make iron metabolism genes of particular interest.

There are several proteins involved in iron metabolism [29]. Genome Wide Association Studies (GWAS) studies have identified polymorphisms of a few genes significantly related to plasma iron levels [30], among them polymorphisms of genes coding for transferrin and its receptor involved in iron transport. Their expression is associated with the regulation of hepcidin involved in the processes of intestinal iron absorption or release of this element by macrophages [29,30]. Considering the information provided about the potential association of iron with sleep patterns, the aim of our study was to establish the frequency of iron metabolism genes polymorphisms and investigate the relationship between genetic variants of the examined genes with sleep pattern disruptions in the Polish persons with ASD.

## 2. Materials and Methods

### 2.1. Study Area

Our study group consisted of 61 Caucasian individuals with ASD, aged 8.26 ± 4.47 years, with male predominance (*n =* 48, 78.68%). A clinical diagnosis of ASD was confirmed by ICD-10 criteria using Autism Diagnostic Observation Schedule-Generic (ADOS, polish version) [31], by a child psychiatrist. Parents of the children were interviewed and a free and guided observation of the child was organized by a doctor. These were supplemented with questionnaires for evaluating sensomotor development. Whole ICD-10 F84 category except Rett syndrome was included. To assess whether there are any differences in particular SNPs prevalence in comparison to neurotypical population, we enrolled a control group of otherwise healthy individuals (without the diagnosis of ASD; *n =* 57, age 8.31 ± 3.82 years). Parents of all persons from the study and control groups were familiarized with the aims and course of the survey and gave written informed consent for their children to participate in the project. The study protocol was positively evaluated by the Ethics Committee of Pomeranian Medical University, Szczecin, Poland (approval No. KB-0012/81/15).

### 2.2. Genotyping

Genotypes were determined by real-time PCR using the Light Cycler^®^ 96 System (Roche Diagnostics, Pleasanton, CA, USA) and TaqMan SNP Genotyping Assays (Life Technologies, Foster City, CA, USA). The identification of the following polymorphisms was carried out: rs10421768 of hepcidin gene (*HAMP*), rs7385804 of transferrin receptor gene (*TFR*2), and rs3811647 and rs1049296 of transferrin gene (*TF*). The assay IDs were C___2604942_10, C___2184545_10, C__27492858_10, and C___7505275_10, respectively. The data were analyzed using Light Cycler software v. 1.0.1 (Roche Diagnostics, Pleasanton, CA, USA). All mutant samples were verified.

### 2.3. Sleep Difficulties

The Athens Insomnia Scale (AIS) was used to identify sleep pattern disruptions. The following quality of sleep determinants were evaluated: difficulties falling asleep, night and early morning awakenings, total sleep time, and well-being during the next day. The survey was parent-administered. The AIS scale ranges from 0 to 3, where “0” stands for no problems of particular sleep behavior, while “3” means that the particular sleep behavior is very disturbed. The parents of autistic individuals were to assign to each of the questions contained in the questionnaire an appropriate score their children experienced at least three times a week during the last month. In original validation studies, it was demonstrated that AIS represents high reliability and validity, and a total score of 6 or more points indicates high probability of insomnia (sensitivity of 93% and specificity of 85%) [32]. The sleep quality was finally evaluated from the cumulative score of all factors and reported as an individual’s sleep outcome.

### 2.4. Statistical Methods

The distribution of continuous variables was verified by the Kolmogorov–Smirnov test (K–S test). The genotype and allele frequencies of investigated SNPs were determined by direct counting. Deviations from Hardy–Weinberg equilibrium (HWE) were assessed with a chi-square (χ^2^) test or an exact test from R package “Hardy Weinberg” v. 1.6.2. (https://cran.r-project.org/web/packages/HardyWeinberg/index.html). Overall differences in genotype distributions between ASD and non-ASD individuals, as well as between ASD cases with and without insomnia, were evaluated using χ^2^ test. For the statistical analysis of the genotypes (under co-dominant, dominant, recessive, and over-dominant genetic models) and alleles association with ASD and insomnia susceptibility, the Fisher’s exact test was applied. The abovementioned statistical analyses were performed with GraphPad Prism v. 8.02 (GraphPad Software Inc., San Diego, CA, USA). A value of *p* < 0.05 was considered statistically significant.

## 3. Results

### 3.1. The Prevalence of Insomnia

As proposed by Soldatos et al. [33], a six-point cut off in AIS was established to be associated with insomnia. Using this criterion, we identified *n =* 32 (57.14%) ASD cases with insomnia within group of *n =* 56 screened individuals from original ASD group. In our study group, the mean number of points obtained in AIS scale was 6.09 ± 3.69 points. We found no differences between delivery mode (*p* > 0.05), breastfeeding (*p* > 0.05), and artificial ventilation (*p* = 0.05) and sleep quality. In the next part of the present study, the ASD group was divided into two groups as cases with no sleep problems (Non-Insomnia), and cases with insomnia (Athens Insomnia Scale ≥ 6).

### 3.2. The Frequency of Iron Metabolism Genes Polymorphisms

We successfully genotyped all available samples and obtained 100% concordance between the genotyped duplicate samples for all analyzed polymorphisms. All investigated SNPs were in HWE in all analyzed groups. The genotype and allele distributions of *TF* rs1049296 C>T, *TF* rs3811647 G>A, *TFR* rs7385804 A>C, and *HAMP* rs10421768 A>G are presented in Table 1. There were no homozygous carriers of the *TF* rs1049296 C>T minor allele in the ASD group; hence, for this SNP, the analysis of differences in the overall distribution of genotypes between ASD and non-ASD individuals as well as between ASD cases with and without insomnia was not feasible. Statistical analyses revealed no significant differences in the overall distribution of genotypes between ASD and non-ASD individuals for three remaining SNPs. Similarly, there were no such differences between ASD cases with and without insomnia. We also found no significant association of all four investigated SNPs with ASD and insomnia at the allele level. In the next step of our analysis, we investigated whether insomnia may be linked to polymorphisms of iron metabolism genes regarding different models of inheritance. We found no such association, as presented in Table 2. Due to absence of homozygous carriers of the *TF* rs1049296: T allele in the group of ASD cases with and without insomnia, the analyses of recessive and co-dominant T/T vs. C/C models were not feasible.

## 4. Discussion

Sleep plays a vital role for every human being, affecting one’s health, well-being, and functioning during the day. It was proved that autonomic nervous system paths are altered during poor sleep patterns, thus introducing an imbalanced psychological condition [34]. Individuals with ASD often experience sleep disorders [35]. Insomnia in autistic children and adolescents is challenging to their families and has been associated with increased maternal distress and parental sleep disruption as well as poor caregiver’s quality of life [35].

In this study, the prevalence of insomnia in ASD cases was 57.14% (*n =* 32). The quality of sleep was finally evaluated from the cumulative score of all factors included in AIS scale and reported as insomnia where the total number of points was at least six. Similar results were provided by other researchers who conducted studies in ASD population, predominantly based on parental reports. Souders et al. [36] analyzed ASD children aged 4–10 years old and identified almost 70% prevalence of sleep problems. According to study by Krakowiak [37], nearly 50% of children with ASD, aged 2–5 years old, experienced at least one sleep problem. The recent report demonstrated that the prevalence of insomnia in ASD varies between 50% and 80% [38]. It was demonstrated that sleep disorders trigger the severity of core behavioral ASD symptoms [39]. It was shown that children with ASD suffering from sleep problems had greater difficulty in relationships with peers, group membership, and daily functioning [40]. Other studies stated that sleep disturbances caused difficulty with concentration and learning, and resulted in increased excitability and reactivity [14].

Insomnia and iron deficiency are widespread problems. According to the World Health Organization (WHO), iron deficiency is the most prevalent nutritional deficiency in industrialized countries, and was reported to be the most widespread problem in the world today [40,41]. Iron is essential to early neurodevelopment and contributes to neurotransmitter production, myelination, and immune functions and has an important role in cognitive behavioral and motor development [6,42]. Iron status is critical for early neurodevelopmental processes that are dysregulated in ASD [43]. It contributes to main biological processes, including: protein expression, oxygen transport, and cell growth. Excessive brain iron deposition plays the most important role in development of brain function disorders and cognitive impairment [44].

As ferritin and iron levels are elevated after iron supplementation, malabsorption is unlikely [28] It was suggested that dietary iron intake in preschool children was two times more inadequate than school-aged children [45]. Thus, it can be speculated that younger children with ASD may be more selective in nutrition and therefore iron deficiency is more common in this age group. Thus, we hypothesized that iron dietary intake deficiency might contribute to the symptoms of ASD and iron deficient children may have more severe manifestations [46].

We examined iron metabolism gene polymorphisms prevalence, namely *TF* rs1049296, *TF* rs3811647, *TFR* rs7385804, and *HAMP* rs10421768, in Polish persons with ASD to investigate their possible role in sleep pattern disruptions. From an epidemiological point of view, it has been shown that no significant differences were found in genotype and allele frequencies between study group and control group for all polymorphisms studied regarding ASD and insomnia incidence (Table 1 and Table 2). While the link between ASD and insomnia has been extensively addressed in the literature, the relationship between prevalence of *TF* rs1049296, *TF* rs3811647, *TFR* rs7385804, and *HAMP* rs10421768 and sleep deprivation is still elusive.

The abovementioned genetic variants were found to be associated with skewed protein expression thus iron load, and as discussed above regarding sleep architecture. For instance, Silva et al. [47] reported that *HAMP* nc.-582A>G polymorphism (rs10421768) predisposed beta-thalassemia patients to elevated serum ferritin levels. Similarly, Bruno et al. [48] reported that the expression of hepcidin may be lowered in persons carrying rs10421768 variant. Indeed, in vitro studies proved that the variant located within E-box may impair transcription factor binding [49]. In addition, Liang et al. [50] hypothesized that the human *HAMP* -582 A>G polymorphism affects hepcidin transcription in macrophages and reported that the G allele of rs10421768 might lead to lower hepcidin production. As far as other polymorphisms are concerned, some GWAS studies confirmed the association between analyzed SNPs and iron level, thus sleep pattern. Tyrac et al. [51] reported that *TF* rs3811647 is significantly associated with TF concentrations in Europeans. Benyamin et al. [52] showed that *TFR*2 rs7385804 is significantly associated with serum iron concentrations in European ancestry. Moreover, McLaren et al. [53] described that *TF* rs3811647 is significantly associated with iron deficiency in American populations. In addition, there is a body of evidence that links few variants within iron metabolism genes with neuropsychiatric diseases. For instance, Wang et al. [54] found that *TF* gene rs1049296 may play a role in Alzheimer’s disease pathogenesis with *TF* C2 as a risk factor. In addition, a study by Greco et al. [55] suggested that the G258S polymorphism within *TF* might be considered a susceptibility factor for Parkinson’s disease.

It must be emphasized that iron deficiency is predominantly of environmental origin in individuals with ASD and sensory modulation disorders. Sensory processing alterations, especially of tactile nature [56], make children refuse taking certain foods, which in combination with behavioral disorders significantly limits repertoire of feeding a child [57]. Consequently, autistic children may be at risk for low iron concentration associated with poor intake of this element [6]. Herndon [58] and others authors [42,43] reported that nearly half of children with ASD had inadequate dietary iron intake. Children with autism often have very restricted food preferences due to smell, taste, texture, or other characteristics of the foods, thus high prevalence of iron deficiency has been reported [6]. Xia et al. [59] found that intake of iron increased with age in 2–9-year-old children with autism. Some studies reported that inadequate dietary iron intake was considered as a cause of iron deficiency, and low iron intake was thought to be associated with food selectivity, which is commonly seen in children with ASD, Tourette’s syndrome (TS), attention deficit hyperactivity disorder (ADHD), and restless legs syndrome (RLS) [6].

Genetic background of iron homeostasis does exist but in light of the available data plays a minor role in iron homeostasis [52,60]. Our study was limited to only molecular analysis, but, to the best of our knowledge, is the only one exploring this issue in ASD individuals. The prevalence of *TF* rs3811647 G>A, *TFR* rs7385804 A>C, and *HAMP*rs10421768 A>G in the persons we analyzed is similar to that evaluated for Europeans previously [61]. According to a database of 1000 genomes [61], the homozygous prevalence for minor allele within *TF* rs1049296 C>T is about 10–15%. In our study, no homozygotes of C2 variant for rs1049296 SNP in the transferrin gene were identified, which might have influenced the results. Indeed, in multiple sclerosis patients, the frequency of this variant was higher when compared to healthy controls but not associated with brain iron level [62]. On the other hand, both positive and negative associations between this SNP and Alzheimer’s disease were found, stating that the statistical power of genetic analyses in neuropsychiatric studies evaluating iron metabolism genes is below the acceptance border. This was also confirmed in our study, predominantly due to low number of participants who entered the study. This is critical for genetic analyses, thus more studies are warranted to verify the results we obtained.

There is also a need to conduct research focusing on relationship of studied polymorphisms and hematological parameters such as: serum iron, hemoglobin, transferring, and total iron blood capacity (TIBC). In our study, we could not analyze these parameters as very few parents gave their consent to collect venous blood from their children. The would definitely bolster the results between iron metabolism and sleep pattern. ASD pathogenesis seems to be of multigenetic background, thus iron homeostasis might be regulated via, e.g., epistatic interaction, as recently demonstrated for the discussed phenotype [63] and iron homeostasis [64]. Likewise, other SNPs with linkage disequilibrium with analyzed variants in *TF*, *TFR,* and *HAM*P genes could be risk factors for iron deficiency in ASD; however, to the best of our knowledge, such analyses have not been conducted so far. In conclusion, hematological parameters analysis could simply elucidate the link among both genes, iron load, and sleep pattern.

Overall, iron is critical for proper neurodevelopment and sleep architecture, thus polymorphism within iron metabolism genes may be linked to these phenotypes. However, in our study, we did not find such a relationship.

## 5. Conclusions

The relationship between prevalence of *TF* rs1049296 C>T, *TF* rs3811647 G>A, *TFR* rs7385804 A>C, and *HAMP* rs10421768 A>G and sleep deprivation is still elusive. Iron deficiency in individuals with ASD may be due to restricted diets and food selectivity, putting them at risk for nutritional deficiencies and high prevalence of iron deficiency thus sleep disorders.

## Figures and Tables

**Table 1:** Genotype and allele distributions of the *TF, TFR* and *HAMP* SNPs in individuals with ASD, without ASD, with ASD and insomnia and with ASD without insomnia.

Polymorphism	ASD (*n* = 61)		Non-ASD (*n* = 57)		*p (*1)	χ^2^ (df)	*p (*2)	OR (95% CI)		ASD + Insomnia (*n* = 32)		ASD + Non-Insomnia (*n* = 24)		*p (*1)	χ^2^ (df)	*p (*2)	OR (95% CI)	
*TF* rs1049296 C>T																		
Genotype, *n*(%)																		
T/T	0	(0.00)	1	(1.75)						0	(0.00)	0	(0.00)					
C/T	20	(32.79)	13	(22.81)	NA	NA	–	–		10	(31.25)	8	(33.33)	NA	NA	–	–	
C/C	41	(67.21)	43	(75.44)						22	(68.75)	16	(66.67)					
Allele, *n* (%)																		
T	20	(16.39)	15	(13.16)	–	–	0.5832	1.29	(0.61–2.69)	10	(15.62)	8	(16.67)	–	–	>0.9999	0.93	(0.36–2.63)
C	102	(83.61)	99	(86.84)	–	–	–	1.00		54	(84.38)	40	(83.33)	–	–	–	1.00	
*TF* rs3811647 G>A																		
Genotype, *n* (%)																		
A/A	5	(8.20)	6	(10.53)						3	(9.37)	2	(8.33)					
G/A	34	(55.74)	26	(45.61)	0.5447	1.2150 (2)	–	–		17	(53.13)	14	(58.34)	0.9275	0.1505 (2)	–	–	
G/G	22	(36.06)	25	(43.86)						12	(37.50)	8	(33.33)					
Allele, *n* (%)																		
A	44	(36.07)	38	(33.33)	–	–	0.6834	1.13	(0.67–1.92)	23	(35.94)	18	(37.50)	–	–	>0.9999	0.94	(0.43–2.09)
G	78	(63.93)	76	(66.67)	–	–	–	1.00		41	(64.06)	30	(62.50)	–	–	–	1.00	
*TFR* rs7385804 A>C																		
Genotype, *n* (%)																		
C/C	10	(16.39)	16	(28.07)						7	(21.87)	3	(12.50)					
A/C	29	(47.54)	25	(43.86)	0.2871	2.4960 (2)	–	–		13	(40.63)	12	(50.00)	0.6234	0.9450 (2)	–	–	
A/A	22	(36.07)	16	(28.07)						12	(37.50)	9	(37.50)					
Allele, *n* (%)																		
C	49	(40.16)	57	(50.00)	–	–	0.1502	0.67	(0.40–1.12)	27	(42.19)	18	(37.50)	–	–	0.6983	1.22	(0.57–2.68)
A	73	(59.84)	57	(50.00)	–	–	–	1.00		37	(57.81)	30	(62.50)	–	–	–	1.00	
*HAMP* rs10421768 A>G																		
Genotype, *n* (%)																		
G/G	4	(6.56)	1	(1.75)						3	(9.37)	1	(4.17)					
A/G	23	(37.70)	17	(29.82)	0.2334	2.9100 (2)	–	–		10	(31.25)	8	(33.33)	0.7553	0.5614 (2)	–	–	
A/A	34	(55.74)	39	(68.43)						19	(59.38)	15	(62.50)					
Allele, *n* (%)																		
G	31	(25.41)	19	(16.67)	–	–	0.1126	1.70	(0.92–3.18)	16	(25.00)	10	(20.83)	–	–	0.6567	1.27	(0.51–3.21)
A	91	(74.59)	95	(83.33)	–	–	–	1.00		48	(75.00)	38	(79.17)	–	–	–	1.00	

χ^2^, chi-square value; 95% CI, 95% confidence interval; ASD, Autism spectrum disorder; df, degrees of freedom; OR, odds ratio; *p* (1), *p* value in two-sided chi-square test; *p* (2), *p* value in two-sided Fisher’s exact test.

**Table 2:** Analysis of the *TF, TFR* and *HAMP* SNPs association with ASD and insomnia (AIS ≥ 6) using genetic inheritance models in individuals with ASD and insomnia (AIS ≥ 6).

Polymorphism	ASD (*n* = 61), *n* (%)		Non-ASD (*n* = 57), *n* (%)		*p*	OR (95% CI)		ASD + Insomnia (*n* = 32), *n*(%)		ASD + Non-Insomnia (*n* = 24), *n*(%)		*p*	OR (95% CI)	
*TF* rs1049296 C>T														
Genetic model														
Co-dominant														
T/T	0	(0.00)	1	(2.27)	>0.9999	0.00	(0.00–9.66)	0	(0.00)	0	(0.00)	NA	NA	
C/C	41	(100.00)	43	(97.73)	–	1.00		22	(100.00)	16	100.00)	–	–	
C/T	20	(32.79)	13	(23.21)	0.3057	1.61	(0.69–3.75)	10	(31.25)	8	(33.33)	>0.9999	0.91	(0.28–2.59)
C/C	41	(67.21)	43	(76.79)	–	1.00		22	(68.75)	16	(66.67)	–	1.00	
Dominant														
T/T+C/T	20	(32.79)	14	(24.56)	0.4164	1.50	(0.65–3.35)	10	(31.25)	8	(33.33)	>0.9999	0.91	(0.28–2.59)
C/C	41	(67.21)	43	(75.44)	–	1.00		22	(68.75)	16	(66.67)	–	1.00	
Recessive														
T/T	0	(0.00)	1	(1.75)	0.4831	0	(0.00–8.41)	0	(0.00)	0	(0.00)	NA	NA	
C/T+C/C	61	(100.00)	56	(98.25)	–	1.00		32	(100.00)	24	(100.00)	–	–	
Over-dominant														
C/T	20	(32.79)	13	(22.81)	0.3050	1.65	(0.71–3.83)	10	(31.25)	8	(33.33)	>0.9999	0.91	(0.28–2.59)
T/T+C/C	41	(67.21)	44	(77.19)	–	1.00		22	(68.75)	16	(66.67)	–	1.00	
*TF* rs3811647 G>A														
Genetic model														
Co-dominant														
A/A	5	(18.52)	6	(19.35)	>0.9999	0.95	(0.27–3.26)	3	(20.00)	2	(20.00)	>0.9999	1.00	(0.17–6.62)
G/G	22	(81.48)	25	(80.65)	–	1.00		12	(80.00)	8	(80.00)	–	1.00	
G/A	34	(60.71)	26	(50.98)	0.3354	1.49	(0.70–3.25)	17	(58.62)	14	(63.64)	0.7780	0.81	(0.28–2.61)
G/G	22	(39.29)	25	(49.02)	–	1.00		12	(41.38)	8	(36.36)	–	1.00	
Dominant														
A/A+G/A	39	(63.93)	32	(56.14)	0.4532	1.39	(0.68–2.86)	20	(62.50)	16	(66.67)	0.7853	0.83	(0.30–2.51)
G/G	22	(36.07)	25	(43.86)	–	1.00		12	(37.50)	8	(33.33)	–	1.00	
Recessive														
A/A	5	(8.20)	6	(10.53)	0.7570	0.76	(0.24–2.83)	3	(9.37)	2	(8.33)	>0.9999	1.14	(0.22–6.82)
G/A+G/G	56	(91.80)	51	(89.47)	–	1.00		29	(90.63)	22	(91.67)	–	1.00	
Over-dominant														
G/A	34	(55.74)	26	(45.61)	0.3570	1.5	(0.71–3.02)	17	(53.13)	14	(58.33)	0.7889	0.81	(0.28–2.26)
A/A+G/G	27	(44.26)	31	(54.39)	–	1.00		15	(46.87)	10	(41.67)	–	1.00	
*TFR* rs7385804 A>C														
Genetic model														
Co-dominant														
C/C	10	(31.25)	16	(50.00)	0.2028	0.46	(0.17–1.29)	7	(36.84)	3	(25.00)	0.6972	1.75	(0.38–7.46)
A/A	22	(68.75)	16	(50.00)	–	1.00		12	(63.16)	9	(75.00)		1.00	
A/C	29	(56.86)	25	(60.98)	0.8316	0.84	(0.36–1.90)	13	(52.00)	12	(57.14)	0.7736	0.81	(0.27–2.82)
A/A	22	(43.14)	16	(39.02)	–	1.00		12	(48.00)	9	(42.86)	–	1.00	
Dominant														
C/C+A/C	39	(63.93)	41	(71.93)	0.4314	0.69	(0.33–1.51)	20	(62.50)	15	(62.50)	>0.9999	1.00	(0.32–2.96)
A/A	22	(36.07)	16	(28.07)	–	1.00		12	(37.50)	9	(37.50)	–	1.00	
Recessive														
C/C	10	(16.39)	16	(28.07)	0.1819	0.50	(0.20–1.23)	7	(21.87)	3	(12.50)	0.4892	1.96	(0.43–7.57)
A/C+A/A	51	(83.61)	41	(71.93)	–	1.00		25	(78.13)	21	(87.50)	–	1.00	
Over-dominant														
A/C	29	(47.54)	25	(43.86)	0.7150	1.16	(0.55–2.32)	13	(40.62)	12	(50.00)	0.5899	0.68	(0.24–1.93)
C/C+A/A	32	(52.46)	32	(56.14)	–	1.00		19	(59.38)	12	(50.00)	–	1.00	
*HAMP* rs10421768 A>G														
Genetic model														
Co-dominant														
G/G	4	(10.53)	1	(2.50)	0.1948	4.59	(0.69–57.36)	3	(13.64)	1	(6.25)	0.6245	2.37	(0.32–32.56)
A/A	34	(89.47)	39	(97.50)	–	1.00		19	(86.36)	15	(93.75)	–	1.00	
A/G	23	(40.35)	17	(30.36)	0.3265	1.55	(0.72–3.26)	10	(34.48)	8	(34.78)	>0.9999	0.99	(0.29–2.88)
A/A	34	(59.65)	39	(69.64)	–	1.00		19	(65.52)	15	(65.22)	–	1.00	
Dominant														
G/G+A/G	27	(44.26)	18	(31.58)	0.1863	1.72	(0.84–3.71)	13	(40.62)	9	(37.50)	>0.9999	1.14	(0.39–3.56)
A/A	34	(55.74)	39	(68.42)	–	1.00		19	(59.38)	15	(62.50)	–	1.00	
Recessive														
G/G	4	(6.56)	1	(1.75)	0.3657	3.93	(0.61–48.83)	3	(9.37)	1	(4.17)	0.6273	2.38	(0.33–32.08)
A/G+A/A	57	(93.44)	56	(98.25)	–	1.00		29	(90.63)	23	(95.83)	–	1.00	
Over-dominant														
A/G	23	(37.70)	17	(29.82)	0.4377	1.42	(0.67–3.17)	10	(31.25)	8	(33.33)	>0.9999	0.91	(0.28–2.59)
G/G+A/A	38	(62.30)	40	(70.18)	–	1.00		22	(68.75)	16	(66.67)	–	1.00	

χ^2^, chi-square value; 95% CI, 95% confidence interval; ASD, Autism spectrum disorder; df, degrees of freedom; OR, odds ratio; *p*, *p* value in two-sided Fisher’s exact test.

## References

[1] American Psychiatric Association (2000). Diagnostic and Statistical Manual of Mental Disorders.

[2] Onore C., Careaga M., Ashwood P. (2012). The role of immune dysfunction in the pathophysiology of autism. Brain. Behav. Immun..

[3] Ratajczak H.V. (2011). Theoretical aspects of autism: Biomarkers-a review. J. Immunol. Toxicol..

[4] American Psychiatric Association (2013). Diagnostic and Statistical Manual of Mental Disorders.

[5] Skonieczna-Żydecka K., Gorzkowska I., Pierzak-Sominka J., Adler G. (2017). The prevalence of autism spectrum disorders in west pomeranian and Pomeranian regions of Poland. J. Appl. Res. Intellect. Disabil..

[6] Hergüner S., Keleşoğlu F.M., Tanıdır C., Cöpür M. (2012). Ferritin and iron levels in children with autistic disorder. Eur. J. Pediatr..

[7] Steyaert J.G., De la Marche W. (2008). What’s new in autism?. Eur. J. Pediatr..

[8] Mannion A., Leader G., Healy O. (2013). An investigation of comorbid psychological disorders, sleep problems, gastrointestinal symptoms and epilepsy in children and adolescents with Autism Spectrum Disorder. Res. Autism Spectr. Disord..

[9] Elrod M.G., Hood B.S. (2015). Sleep differences among children with autism spectrum disorders and typically developing peers: A meta-analysis. J. Dev. Behav. Pediatr..

[10] Dahl R.E. (2004). Adolescent brain development: A period of vulnerabilities and opportunities. Ann. N. Y. Acad. Sci..

[11] Cohen S., Conduit R., Lockley S.W., Rajaratnam S.M., Cornish K.M. (2014). The relationship between sleep and behavior in autism spectrum disorder (ASD): A review. J. Neurodev. Disord..

[12] Schreck K.A., Mulick J.A., Smith A.F. (2004). Sleep problems as possible predictors of intensified symptoms of autism. Res. Dev. Disabil..

[13] Capdevila O.S., Kheirandish-Gozal L., Dayyat E., Gozal D. (2008). Pediatric obstructive sleep apnea: Complications, management, and long-term outcomes. Proc. Am. Thorac. Soc..

[14] Richdale A.L., Schreck K.A. (2009). Sleep problems in autism spectrum disorders: Prevalence, nature, & possible biopsychosocial aetiologies. Sleep Med. Rev..

[15] Ebisawa T. (2007). Circadian rhythms in the CNS and peripheral clock disorders: human sleep disorders and clock genes. J. Pharm. Sci..

[16] Bourgeron T. (2007). The possible interplay of synaptic and clock genes in autism spectrum disorders. Cold Spring Harb. Symp. Quart. Biol..

[17] Nicholas B., Rudrasingham V., Nash S., Kirov G., Owen M.J., Wimpory D.C. (2007). Association of per1 and Npas2 with autistic disorder: Support for the clock genes/social timing hypothesis. Mol. Psychiatry.

[18] Beltrán-Navarro B., Matute E., Vásquez-Garibay E., Zarabozo D. (2012). Effect of chronic iron deficiency on neuropsychological domains in infants. J. Child. Neurol..

[19] Beard J.L. (2000). Effectiveness and strategies of iron supplementation during pregnancy. Am. J. Clin. Nutr..

[20] McCann J.C., Ames B.N. (2007). An overview of evidence for a causal relation between iron deficiency during development and deficits in cognitive or behavioral function. Am. J. Clin. Nutr..

[21] Erikson K.M., Jones B.C., Hess E.J., Zhang Q., Beard J.L. (2001). Iron deficiency decreases dopamine D1 and D2 receptors in rat brain. Pharmacol. Biochem. Behav..

[22] Dzirasa K., Ribeiro S., Costa R., Santos L.M., Lin S.C., Grosmark A., Sotnikova T.D., Gainetdinov R.R., Caron M.G., Nicolelis M.A. (2006). Dopaminergic control of sleep-wakestates. J. Neurosci..

[23] Dauvilliers Y., Winkelmann J. (2013). Restless legs syndrome: Update on pathogenesis. Curr. Opin. Pulm. Med..

[24] Allen R. (2004). Dopamine and iron in the pathophysiology of restless legs syndrome (RLS). Sleep Med..

[25] Youssef J., Singh K., Huntington N., Becker R., Kothare S.V. (2013). Relationship of serum ferritin levels to sleep fragmentation and periodic limb movements of sleep on polysomnography in Autism Spectrum Disorders. Pediatr. Neurol..

[26] Connor J.R., Boyer P.J., Menzies S.L., Dellinger B., Allen R.P., Ondo W.G., Earley C.J. (2003). Neuropathological examination suggests impaired brain iron acquisition in restless legs syndrome. Neurology.

[27] Earley C.J., Connor J.R., Beard J.L., Malecki E.A., Epstein D.K., Allen R.P. (2000). Abnormalities in CSF concentrations of ferritin and transferrin in restless legs syndrome. Neurology.

[28] Dosman C.F., Brian J.A., Drmic I.E., Senthilselvan A., Harford M.M., Smith R.W., Sharieff W., Zlotkin S.H., Moldofsky H., Roberts S.W. (2007). Children with autism: Effect of iron supplementation on sleep and ferritin. Pediatr. Neurol..

[29] Mazur G., Wróbel T., Nosol J. (2005). Rola hepcydyny w regulacji homeostazy żelaza. Acta Haematol. Pol..

[30] An P., Wu Q., Wang H., Guan Y., Mu M., Liao Y., Zhou D., Song P., Wang C., Meng L. (2012). TMPRSS6, but not TF, TFR2 or BMP2variants are associated with increased risk of iron-deficiency anemia. Hum. Mol. Genet..

[31] Chojnicka I., Płoski R. (2012). Polish version of the ADOS (Autism Diagnostic Observation Schedule-Generic). Psychiatr. Pol..

[32] Tobaldini E., Costantino G., Solbiati M., Cogliati C., Kara T., Nobili L., Montano N. (2017). Sleep, sleep deprivation, autonomic nervous system and cardiovascular diseases. Neurosci. Biobehav. Rev..

[33] Soldatos C.R., Dikeos D.G., Paparrigopoulos T.J. (2003). The diagnostic validity of the Athens Insomnia Scale. J. Psychosom. Res..

[34] Thorisson G.A., Smith A.V., Krishnan L., Stein L.D. (2005). The International HapMap Project Web site. Genome Res..

[35] Maras A., Schroder C.M., Malow B.A., Findling R.L., Breddy J., Nir T., Shahmoon S., Zisapel N., Gringras P. (2018). Long-Term Efficacy and Safety of Pediatric Prolonged-Release Melatonin for Insomnia in Children with Autism Spectrum Disorder. J. Child. Adol. Psychop..

[36] Souders M.C., Mason T.B., Valladares O., Bucan M., Levy S.E., Mandell D.S., Weaver T.E., Pinto-Martin J. (2009). Sleep behaviors and sleep quality in children with autism spectrum disorders. Sleep.

[37] Krakowiak P., Goodlin-Jones B., Hertz-Picciotto I., Croen L.A., Hansen R.L. (2008). Sleep problems in children with autism spectrum disorders, developmental delays, and typical development: A population based study. J. Sleep Res..

[38] Cuomo B.M., Vaz S., Lee E.A.L., Thompson C., Rogerson J.M., Falkmer T. (2017). Effectiveness of sleep-based interventions for children with autism spectrum disorder: A meta-synthesis. Pharmacotherapy.

[39] Sikora D.M., Johnson K., Clemons T., Katz T. (2012). The relationship between sleep problems and daytime behavior in children of different ages with autism spectrum disorders. Pediatrics.

[40] Ming X., Gordon E., Kang N., Wagner G.C. (2008). Use of clonidine in children with autism spectrum disorders. Brain Dev..

[41] Bener A., Khattab A.O., Bhugra D., Hoffmann G.F. (2017). Iron and vitamin D levels among autism spectrum disorders children. Ann. Afr. Med..

[42] Beard J.L., Dawson H., Pinero D.J. (1996). Iron metabolism: A comprehensive review. Nutr. Rev..

[43] Schmidt R.J., Tancredi D.J., Krakowiak P., Hansen R.L., Ozonoff S. (2014). Maternal Intake of Supplemental Iron and Risk of Autism Spectrum Disorder. Am. J. Epidemiol..

[44] Chen L., Wei X., Liu C., Li C., Zhou Z. (2018). Brain iron deposition in primary insomnia. An in vivo susceptibility-weighted imaging study. Brain Behav..

[45] Gajowiak A., Styś A., Starzyński R.R., Bednarz A., Lenartowicz M., Staroń R., Lipiński P. (2016). Mice overexpressing both non-mutated human SOD1 and mutated SOD1G93A genes: A competent experimental model for studying iron metabolism in amyotrophic lateral sclerosis. Front. Mol. Neurosci..

[46] Juneja M., Jain R., Singh V., Mallika V. (2010). Iron Deficiency in Indian Children with Attention Deficit. Hyperactivity Disorder. Indian Pediatr..

[47] Silva B., Pita L., Gomes S., Goncalves J., Faustino P. (2014). The hepcidin gene promoter nc.-1010C > T; −582A > G haplotype modulates serum ferritin in individuals carrying the common H63D mutation in HFE gene. Ann. Hematol..

[48] Bruno F., Bonalumi S., Camaschella C., Ferrari M., Cremonesi L. (2010). The -582A > G variant of the HAMP promoter is not associated with high serum ferritin levels in normal subjects. Haematologica.

[49] Parajes S., Gonzalez-Quintela A., Campos J., Quinteiro C., Dominguez F., Loidi L. (2010). Genetic study of the hepcidin gene (HAMP) promoter and functional analysis of the c.-582A > G variant. BMC Gene.

[50] Liang L., Liu H., Yue J., Liu L.R., Han M., Lou L.L., Zhao Y.L., Xiao H. (2017). Association of Single-Nucleotide Polymorphism in the Hepcidin Promoter Gene with Susceptibility to Extrapulmonary Tuberculosis. Genet. Test. Mol. Biomark..

[51] de Tayrac M., Roth M.P., Jouanolle A.M., Coppin H., le Gac G., Piperno A., Férec C., Pelucchi S., Scotet V., Bardou-Jacquet E. (2015). Genome-wide association study identifies TF as a significant modifier gene of iron metabolism in HFE hemochromatosis. J. Hepatol..

[52] Benyamin B., Esko T., Ried J.S., Radhakrishnan A., Vermeulen S.H., Traglia M., Gögele M., Anderson D., Broer L., Podmore C. (2014). Novel loci affecting iron homeostasis and their effects in individuals at risk for hemochromatosis. Nat. Commun..

[53] McLaren C.E., Garner C.P., Constantine C.C., McLachlan S., Vulpe C.D., Snively B.M., Gordeuk V.R., Nickerson D.A., Cook J.D., Leiendecker-Foster C. (2011). Genome-wide association study identifies genetic loci associated with iron deficiency. PLoS ONE.

[54] Wang Y., Xu S., Liu Z., Lai C., Xie Z., Zhao C., Wei Y., Bi J.Z. (2013). Meta-analysis on the association between the TF gene rs1049296 and AD. Can. J. Neurol. Sci..

[55] Greco V., De Marco E.V., Rocca F.E., Annesi F., Civitelli D., Provenzano G., Tarantino P., Scornaienchi V., Pucci F., Salsone M. (2011). Association study between four polymorphisms in the HFE, TF and TFR genes and Parkinson’s disease in Southern Italy. Neurol. Sci..

[56] Fernández-Andrés M.I., Pastor-Cerezuela G., Sanz-Cervera P., Tárraga-Mínguez R. (2015). A comparative study of sensory processing in children with and without Autism Spectrum Disorder in the home and classroom environment. Res. Dev. Disabil..

[57] Cermak S.A., Curtin C., Bandini L.G. (2010). Food selectivity and sensory sensitivity in children with autism spectrum disorders. J. Am. Diet. Assoc..

[58] Herndon A.C., DiGuiseppi C., Johnson S.L., Leiferman J., Reynold A. (2009). Does nutritional intake differ between children with autism spectrum disorders and children with typical development?. J. Autism Dev. Disord..

[59] Xia W., Zhou Y., Sun C., Wang J., Wu L. (2010). A preliminary study on nutritional status and intake in Chinese children with autism. Eur. J. Pediatr..

[60] Stickel F., Buch S., Zoller H., Hultcrantz R., Gallati S., Österreicher C., Finkenstedt A., Stadlmayr A., Aigner E., Sahinbegovic E. (2014). Evaluation of genome-wide loci of iron metabolism in hereditary hemochromatosis identifies PCSK7 as a host risk factor of liver cirrhosis. Hum. Mol. Genet..

[61] 1000 Genomes Project. 1000Genomes.org.

[62] Hagemeier J., Ramanathan M., Schweser F., Dwyer M.G., Lin F., Bergsland N., Weinstock-Guttman B., Zivadinov R. (2018). Iron-related gene variants and brain iron in multiple sclerosis and healthy individuals. Neuroimage Clin..

[63] Mitra I., Lavillaureix A., Yeh E., Traglia M., Tsang K., Bearden C.E., Rauen K.A., Weiss L.A. (2017). Reverse pathway genetic approach identifies epistasis in autism spectrum disorders. PLoS Genet..

[64] Benyamin B., McRae A.F., Zhu G., Gordon S., Henders A.K., Palotie A., Peltonen L., Martin N.G., Montgomery G.W., Whitfield J.B. (2009). Variants in TF and HFE Explain ∼40% of Genetic Variation in Serum-Transferrin Levels. Am. J. Hum. Genet..

